# African Swine Fever Virus Bearing an I226R Gene Deletion Elicits Robust Immunity in Pigs to African Swine Fever

**DOI:** 10.1128/JVI.01199-21

**Published:** 2021-11-09

**Authors:** Yanyan Zhang, Junnan Ke, Jingyuan Zhang, Jinjin Yang, Huixian Yue, Xintao Zhou, Yu Qi, Rongnian Zhu, Faming Miao, Qian Li, Fei Zhang, Ying Wang, Xun Han, Lijuan Mi, Jinmei Yang, Shoufeng Zhang, Teng Chen, Rongliang Hu

**Affiliations:** a Changchun Veterinary Research Institute, Chinese Academy of Agricultural Sciences, Changchun, China; b College of Animal Science and Technology, Jilin Agriculture University, Changchun, People’s Republic of China; Emory University School of Medicine

**Keywords:** African swine fever virus, I226R gene, deletion, vaccine candidate

## Abstract

African swine fever (ASF) is a severe hemorrhagic infectious disease in pigs caused by African swine fever virus (ASFV), leading to devastating economic losses in epidemic regions. Its control currently depends on thorough culling and clearance of the diseased and surrounding suspected pigs. An ASF vaccine has been extensively explored for years worldwide, especially in hog-intensive areas where it is highly desired, but it is still unavailable for numerous reasons. Here, we report another ASF vaccine candidate, named SY18ΔI226R, bearing a deletion of the I226R gene with a replacement of an enhanced green fluorescent protein (eGFP) expression cassette at the right end of the viral genome. This deletion results in the complete loss of virulence of SY18 as the gene-deleted strain does not cause any clinical symptoms in all pigs inoculated with a dosage of either 10^4.0^ or 10^7.0^ 50% tissue culture infective doses (TCID_50_). Apparent viremia with a gradual decline was monitored, while virus shedding was detected only occasionally in oral or anal swabs. ASFV-specific antibody appeared at 9 days postinoculation. After intramuscular challenge with its parental strain ASFV SY18 at 21 days postinoculation, all the challenged pigs survived, without obvious febrile or abnormal clinical signs. No viral DNA could be detected upon the dissection of any tissue when viremia disappeared. These results indicated that SY18ΔI226R is safe in swine and elicits robust immunity to virulent ASFV infection.

**IMPORTANCE** Outbreaks of African swine fever have resulted in devastating losses to the swine industry worldwide, but there is currently no commercial vaccine available. Although several vaccine candidates have been reported, none has been approved for use for several reasons, especially ones concerning biosafety. Here, we identified a new undescribed functional gene, I226R. When deleted from the ASFV genome, the virus completely loses its virulence in swine. Importantly, pigs infected with this gene-deleted virus were resistant to infection by intramuscular challenge with 10^2.5^ or 10^4.0^ TCID_50_ of its virulent parental virus. Furthermore, the nucleic acid of the gene-deleted virus and its virulent parental virus was rarely detected from oral or anal swabs. Viruses could not be detected in any tissues after necropsy when viremia became negative, indicating that robust immunity was achieved. Therefore, SY18ΔI226R is a novel, ideal, and efficacious vaccine candidate for genotype II ASF.

## INTRODUCTION

African swine fever (ASF) is a highly contagious febrile infectious disease in all species of pigs, including domestic pigs (Sus scrofa *domesticus*) and wild boar (Sus scrofa) (1), caused by African swine fever virus (ASFV), a large and complex double-stranded DNA virus and the only member of the genus *Asfivirus* in the family *Asfarviridae* (2, 3). ASFV is usually maintained by warthogs (Phacochoerus africanus) and soft ticks (*Ornithodoros* spp.) in African countries (1), and it is transcontinentally transmitted and spread by infected pork products or contaminated tissues, items, and vehicles (4–7). Depending on the difference in virulence, ASF manifestations include peracute, acute, subacute, chronic, and subclinical or inapparent infection forms, with 90 to 100% mortality to no clinical signs or even no lesions (8, 9). In ASF epidemic regions, the pig industry has encountered great economic losses. Since the occurrence of ASF in China in August 2018 (10), it has also been reported in several other Asian countries such as Vietnam (11), Mongolia (12), South Korea (13), and other countries (14). ASF is a notifiable infectious disease to the World Animal Health Organization (OIE). Thousands of outbreaks in Asia have been reported to the OIE, and this resulted in the culling of dozens of millions of pigs (https://rr-asia.oie.int/en/projects/asf/). Underreported cases often seen in remote areas with poor veterinary public hygiene and disease detection systems further promote the spread of the disease. In other areas, the pig industry is being threatened all the time by the disease in the background of active global trade (15).

At present, the control measures for ASF include strict quarantine at borders, thorough biosafety management in farms, culling of animals that contracted/were exposed to the disease, and movement restrictions in affected areas. However, disease control has proven to be a challenge, especially in pig breeding countries where pig populations are condensed and biosafety conditions are poor. At present, many countries in Asia have been affected by the disease (16). Efforts have been made to develop a safe and effective vaccine, but it seems that all the candidates are still in the laboratory. Recently, the gene-deleted live vaccine candidates ASFV-G-ΔI177L (17), SY18ΔMGF/CD2v (18), and ASFV HLJ18-7GD (19), which possess single or multiple gene deletions, were reported to provide complete protection and whole-life safety for inoculated fattening pigs. However, long-term biosafety in the field is still under extensive evaluation. Concerns about further mutations and recombinations among the vaccine strains and the epidemic virus are increasing among professionals, administrators, and farmers. However, according to past experiences and understanding, gene-deleted live vaccines are still the most promising vaccine for ASF, as both naturally attenuated and artificially attenuated ASFVs exhibited high potency in stimulating stronger protection against virulent ASFV challenge than the other vaccines or vaccine combinations. For example, deletions of MGF360/MGF505, UK, CD2v, 9GL, I177L, DP148R, and L7L-L11L reduced the virulence of the original isolates to some extent and protected pigs from challenges in their vaccination trials (17, 20–26).

The role of the ASFV I226R gene has not been reported previously. It is a conserved gene with only minor variations in amino acids, even in the nucleotide sequences of all genotypes of ASFV according to our primary analysis, except for Malawi Lil-20/1, which possesses several amino acid mutations (27). Natural deletion of this gene has never occurred in all reported isolates. The conserved sequence and its stable existence in the virus genome give us a clue that it might be necessary for virus dispersion or virulence maintenance. To explore the preliminary function of this gene and to find out its importance for the virulence of ASFV, we deleted I226R by replacing it with an enhanced green fluorescent protein (GFP) expression cassette and performed inoculation and challenge experiments in swine. The results showed that animals inoculated with virus lacking the I226R gene remained clinically normal and developed a strong virus-specific antibody response; importantly, deletant strain ASFV SY18ΔI226R-inoculated pigs were completely protected when challenged with its parental strain SY18.

## RESULTS

### I226R is highly conserved among ASFVs.

The I226R gene is located at the variable region of the 3′ end of the left strand of the ASFV genome between the I267L and I243L genes. As for the ASFV SY18 isolate, the I226R gene is located at bp 170634 to 171314 of the genome. The open reading frame (ORF) of the I226R gene consists of 681 bp and encodes 266 amino acids, and the molecular weight (MW) of the peptide/protein is 27 kDa.

**FIG 1:**
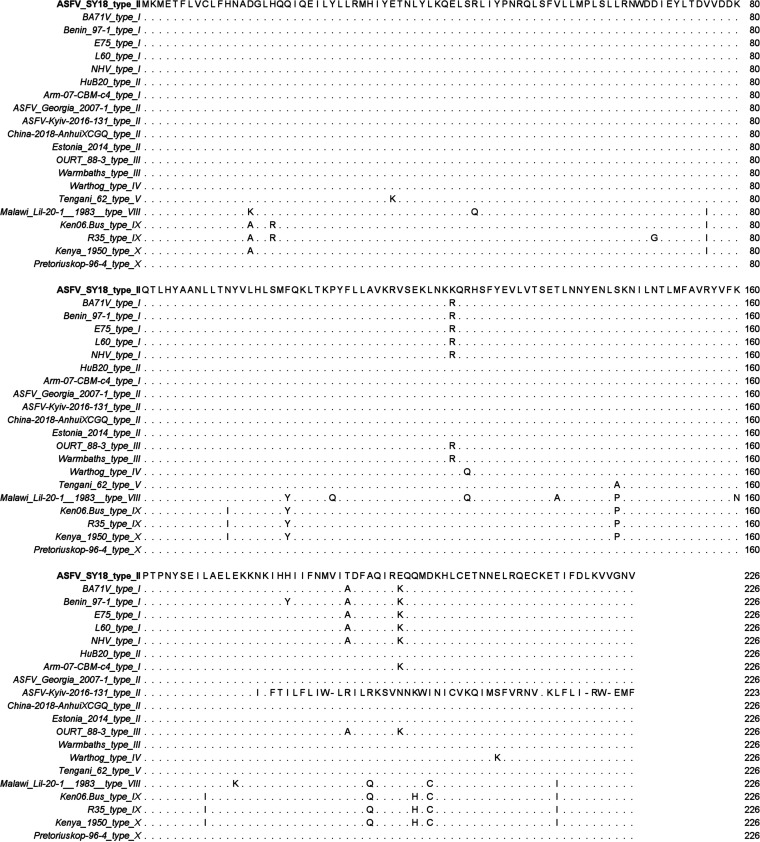
Multiple-sequence alignments of the pI226R amino acid residues among different ASFV isolates. The similarity of amino acids among isolates ranges from 77.9 to 100%. “.” represents the same amino acids. Capital letters represent different amino acids. “|” represents absent amino acids.

The most divergent pI226R sequences among the different ASFVs are shown in Fig. 1. Twenty-one pI226R amino acid sequences among different genotype isolates were downloaded from the NCBI (accession no. U18466.2, NC_044956.1, NC_044958.1, NC_044941.1, NC_044943.1, MH766894.2, MW521382.1, LR881473.1, NC_044959.2, MN194591.1, MK128995.1, LS478113.1, NC_044957.1, AY261365.1, AY261366.1, AY261364.1, AY261361.1, NC_044946.1, MH025920.1, AY261360.1, and AY261363.1). A nucleotide BLAST (https://blast.ncbi.nlm.nih.gov/Blast.cgi) analysis showed that the homology of the I226R gene among all isolates ranged from 94 to 100% as the homology of the corresponding amino acids ranged from 77.9 to 100%, indicating that pI226R is a highly conserved protein. None of the isolates reported in GenBank so far have lacked the gene, and no similar genes/peptides were found in GenBank and protein databases except for ASFVs. Hence, the function of pI226R is not easy to predict, and it is difficult to judge whether the diverse virulence is related to I226R mutations. The length of this protein in all isolates is 226 amino acid residues, except for ASFV/Kyiv/2016/131, which has a nucleotide mutation leading to early termination and truncation from 226 amino acids to 177 amino acids.

### I226R is transcribed at the late stage of infection.

**FIG 2:**
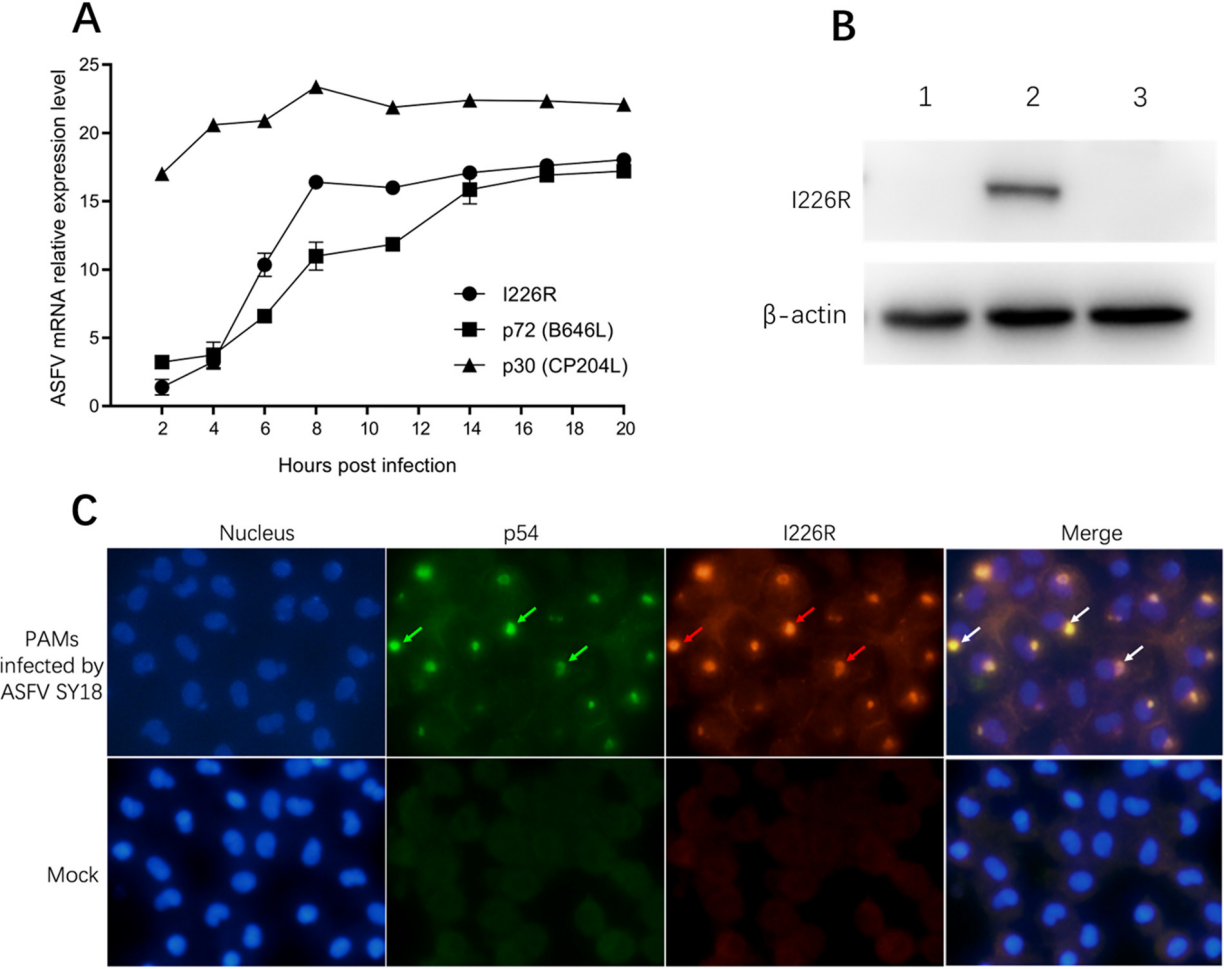
Expression characteristics of pI226R. (A) Relative mRNA expression levels of B646L, CP204L, and I226R at different stages of infection. The mRNA expression level of CP204L remained high since infection, while the mRNA levels of B646L and I226R had similar expression trends and increased continually since infection. (B) The specificity of rabbit anti-pI226R polyclonal antibody was examined by Western blotting, with actin used as an internal reference. Lane 1, PAMs infected by ASFV SY18ΔI226R; lane 2, PAMs infected by ASFV SY18; lane 3, cultured PAMs as mock. (C) Subcellular location of pI226R in PAMs. pI226R is visualized in ASFV-infected PAMs by indirect immunofluorescence staining. DAPI staining (blue) shows the nucleus, TRITC immunostaining (red) shows pI226R (red arrows), and FITC immunostaining shows p54 (green arrows). pI226R and p54 were overlaid (white arrows) near the nucleus (the magnification of all scopes is 200×).

We compared the transcription phases of 3 different viral proteins against cellular glyceraldehyde-3-phosphate dehydrogenase (GAPDH) expression using reverse transcription-quantitative PCR (RT-qPCR). The results showed that CP204L (p30) and B646L (p72), as previously reported, are early- and late-expressed viral proteins, respectively. The transcription of I226R was limited within the first 6 h postinoculation (hpi) and then could be detected throughout the remaining infection cycle. More importantly, I226R possesses a transcription curve similar to that of the late protein B646L, as shown in Fig. 2A, and hence, I226R was also considered a late-expression gene/protein.

### pI226R is localized in the cytoplasm at the viral factory.

The rabbit anti-pI226R polyclonal antibody had a significant specificity target of pI226R (Fig. 2B). The subcellular localization of pI226R was visualized by an immunofluorescence assay to target ASFV SY18-infected pulmonary alveolar macrophages (PAMs) with the rabbit-specific I226R antibody. The nucleus was stained by 4′,6-diamidino-2-phenylindole (DAPI), which is presented in blue. It was shown that the rabbit anti-pI226R serum reacted with pI226R and presented as red fluorescence, emitted by tetramethyl rhodamine isothiocyanate (TRITC)-labeled goat anti-rabbit IgG. This staining result is colocalized by p54 staining. Both pI226R and p54 were observed outside the nucleus and located in the “viral factory” in the cytoplasm (Fig. 2C).

### Generation of gene-deleted SY18ΔI226R.

**FIG 3:**
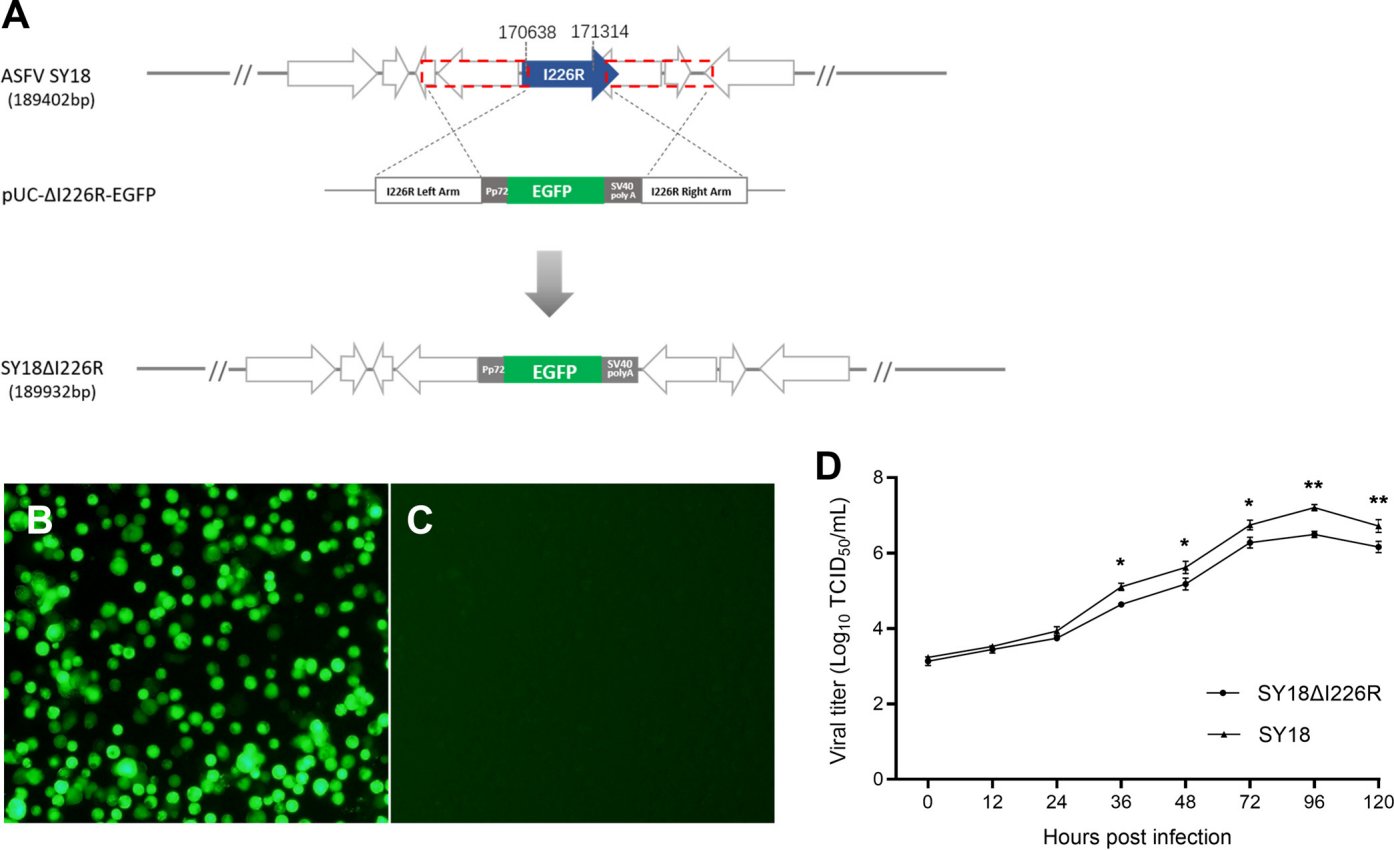
Construction and identification of the I226R gene deletion strain SY18ΔI226R. (A) Schematic representation of SY18ΔI226R construction. The location of the I226R gene was replaced with the eGFP cassette via homologous recombination between pUC-ΔI226R-EGFP and ASFV SY18 genomic DNA *in vitro*. SV40, simian virus 40. (B and C) Purified I226R gene deletion strain SY18ΔI226R expressed eGFP and the cell control. (D) *In vitro* replication kinetics of gene-deleted ASFV SY18ΔI226R compared to ASFV SY18. Three repeats of PAMs were infected with each virus at an MOI of 0.01, harvested at different time points, and assayed on a 96-well plate. The viral titers are exhibited as log_10_ TCID_50_ per milliliter. (*, *P < *0.05; **, *P < *0.01).

The strategy and flow chart to construct I226R gene-deleted ASFV are shown in Fig. 3A. As the ORFs of I226R and I243L are located on different strands of the viral genomic DNA, the ends of the I226R and I243L ORFs share 4 bp. It is necessary to avoid destroying the I243L ORF while deleting the I226R ORF. Therefore, 673 bp were deleted instead of 681 bp of I226R to protect the integrity of the I243L ORF. After transfection and infection of PAMs, cell foci with fluorescence were taken out. After rounds of limiting dilution, the viruses were purified and named SY18ΔI226R (Fig. 3B and C). Conventional PCR on the purified virus using differential primers produced no band of 492 bp in size.

Next-generation sequencing (NGS) was performed on the purified virus, and the results showed that the full-length modified recombinant SY18ΔI226R genome was 189,932 bp. A 673-bp deletion was achieved, and a 1,193-bp eGFP cassette was inserted as designed. Surprisingly, except for the deleted fragment, three mutations occurred, compared to the original SY18 genome sequences. One is in the MGF360-11L ORF, the absence of 1 base C, leading to the premature termination of translation and truncating the amino acids from 353 to 89. Another is the insertion of 7 bases between E423R and E301R. The third is a single-base mutation in the E199L ORF, which resulted in the conversion of glutamic acid (Glu) to valine (Val) (Table 1). All these variations were confirmed by Sanger sequencing.

**TABLE 1:** Genome sequence variations of SY18ΔI226R^*a*^

Site (bp)	Gene(s)	Base mutation	aa change	Peptide length (aa)
27292	MGF360-11L	Delete C	Encoding shift	353→89
164262	Spacer regions of ORFs between E423R and E301R	Insert AAAGTCT	/	/
166105	E199L	T→A	Glu→Val	199

a /, no application; aa, amino acid.

### Deletion of the I226R gene does not affect the replication of SY18ΔI226R *in vitro*.

The growth characteristics of SY18ΔI226R were compared with those of its virulent parental virus SY18 using their titers in PAMs at different time points. As shown in Fig. 3D, SY18ΔI226R has decelerated growth kinetics compared to those of the parental virus. The multiplication of SY18ΔI226R at 12 hpi became slower than that of the parent strain and maintained the trend from 24 to 120 hpi. The cytopathic effect (CPE) in SY18ΔI226R-infected cells appeared earlier and was observed to be more apparent than with its parental virus SY18. Earlier cell abscission was also observed. Curiously, the titers of both viruses began to drop when cultured for 96 hpi, and significantly, the titer of SY18ΔI226R was 4.3 to 7.9 (10^0.63^ to 10^0.9^) times lower than that of its parental strain SY18 from 36 hpi to 120 hpi.

### SY18ΔI226R loses virulence in domestic pigs.

**FIG 4:**
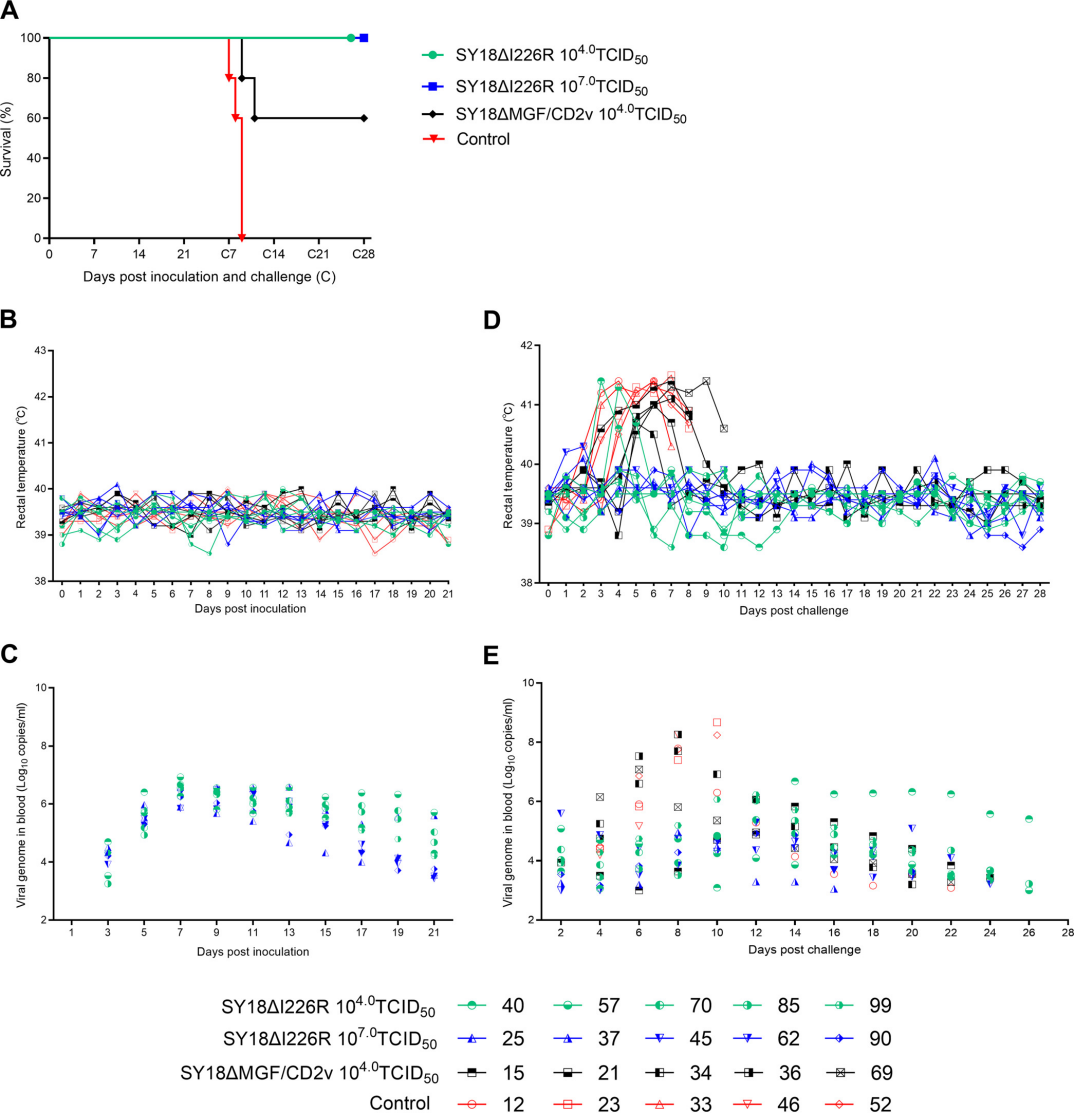
Results for survival rate, temperature, and viremia after inoculation and challenge. (A) Survival outcomes of pigs after i.m. inoculation and challenge. (B) Rectal temperature of the pigs inoculated with SY18ΔI226R at 10^4.0^ and 10^7.0^ TCID_50_, SY18ΔMGF/CD2v at 10^4.0^ TCID_50_, and PBS with the same volume as the one indicated above. (C) Viremia (shown as viral genome copies) in all groups of pigs after inoculation. (D) Rectal temperatures of the four groups of pigs after challenge. Shown are the temperature variations in groups that received 10^4.0^ TCID_50_ SY18ΔI226R, 10^7.0^ TCID_50_ SY18ΔI226R, 10^4.0^ TCID_50_ SY18ΔMGF/CD2v, and the control after ASFV SY18 challenge. (E) Viremia in pigs after i.m. challenge. Genomic copy levels in the blood of the pigs inoculated with SY18ΔMGF/CD2v, SY18ΔI226R, ASFV SY18, and the control are shown. (The equation is *y* = −3.34*x* + 40.1 in the probe-based qPCR, where *y* is the cycle threshold [*C_T_*] value and *x* is log_10_ copies per milliliter. The amplification efficiency [*E*] is 99.2%, and the correlation coefficient [*R*^2^] is 1.)

Twenty Landrace pigs were randomly divided into 4 groups. The first 3 groups were inoculated with 10^4.0^ 50% tissue culture infective doses (TCID_50_) of SY18ΔI226R, 10^7.0^ TCID_50_ of SY18ΔI226R, and 10^4.0^ TCID_50_ of SY18ΔMGF/CD2v, respectively, and another group was set as the challenge control. During the whole observation period after inoculation and before challenge, all 3 inoculated groups of pigs remained healthy and presented normal appetite, behavior, and excretion, without any clinical abnormalities, and no illness or death occurred (Fig. 4A), compared to the controls. The variations in rectal temperature for each individual are illustrated in Fig. 4B. Although the rectal temperatures varied throughout the observation period in all groups, all were kept relatively stable within the physiological range, from the lowest temperature of 38.6°C to the highest temperature of 40.1°C. Viremia in each group is illustrated in Fig. 4C. Apparent viremia was produced on day 3 postinoculation in the SY18ΔI226R-inoculated groups, peaked on days 8 to 12, and gradually declined afterwards. No accompanying clinical signs or physical abnormalities with viremia were observed and detected as described above. All this indicates that the deletion of I226R results in a complete loss of virulence, while viremia in the sera of the SY18ΔMGF/CD2v-inoculated pigs was not detected after inoculation, which is consistent with our previous results (18).

### SY18ΔI226R provided 100% protection against homologous virus challenge.

After 21 days postinoculation (dpi), all 4 groups of pigs were challenged with ASFV SY18. The rectal temperatures showed that the pigs in the control group had fever 3 days after the challenge (Fig. 4D) and gradually presented clinical signs like inappetence, depression, and reluctance to stand. All pigs were euthanized *in extremis* using pentobarbital at 10 days (Fig. 4A). The pigs inoculated with SY18ΔMGF/CD2v showed high fever from 3 to 7 days postchallenge (dpc), which lasted 2 to 6 days postchallenge in different individuals. Two out of five pigs that developed serious clinical signs were euthanized at 8 dpc and 11 dpc, and 3/5 pigs recovered from the challenge (Fig. 4A), while in the SY18ΔI226R inoculation group, regardless of low or high doses, all pigs survived the challenge (Fig. 4A), and only 2/5 pigs inoculated with a low dose of SY18ΔI226R showed sign of fever for 2 days and rapidly recovered to normal. The clinical outcomes are outlined in Table 2 and Fig. 4A. Viremia was detected, and the results are shown in Fig. 4E. It was shown that after challenge, all pigs in each group presented obvious viremia, which might be caused by the challenge virus SY18. All the challenge control pigs were euthanized when they were moribund within 10 days after challenge. Viremia in the SY18ΔMGF/CD2v-vaccinated pigs lasted for more than 4 weeks, while viremia in pigs inoculated with SY18ΔI226R was cleared around 4 to 5 weeks after challenge.

**TABLE 2:** Clinical presentations and survival outcomes of pigs after challenge^*a*^

Group	ASFV SY18 challenge dose (TCID_50_)	Mean value for fever parameter (SD)			No. of survivors/no. of pigs challenged	Mean no. of days to death (SD)
		Day of onset	Duration (no. of days)	Highest rectal temp (°C)		
10^4.0^ TCID_50_ SY18ΔI226R	10^2.5^	3.5 (0.71)	2.0 (0.00)	41.4	5/5	/
10^7.0^ TCID_50_ SY18ΔI226R	10^4.0^	/	/	/	5/5	/
10^4.0^ TCID_50_ SY18ΔMGF/CD2v	10^2.5^	5.2 (0.45)	3.8 (2.28)	41.4	3/5	9.0 (1.41)
Challenge control	10^2.5^	3.8 (0.84)	4.4 (0.55)	41.6	0/5	7.4 (0.90)

a /, absence.

### Occasional shedding occurs through the oral or anal route after SY18ΔI226R inoculation and ASFV SY18 challenge.

**FIG 5:**
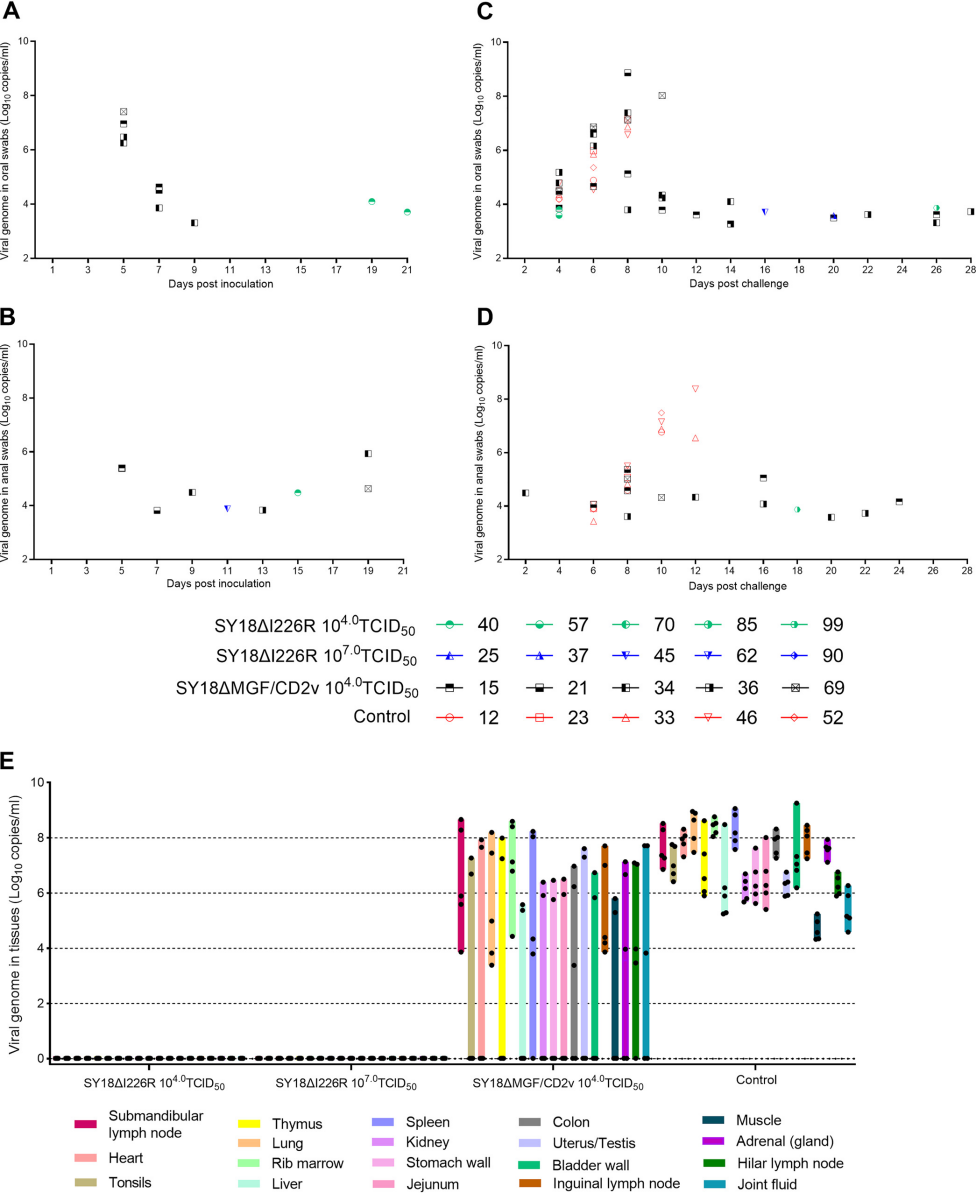
Attenuated and virulent ASFV genome DNA detection in oral swabs, anal swabs, and tissues of pigs in the four groups. (A and B) Attenuated ASFV genomic copies (log_10_ copies per milliliter) detected by amplification of the p72 gene on oral and anal swabs before challenge. (C and D) ASFV genomic copies detected by amplification of the p72 gene on oral and anal swabs after challenge. (E) ASFV genomic copies detected by amplification of the I226R gene in the tissues of inoculated pigs after necropsy. Different bar colors represent different tissues, and the bar length is correlated with the maximum and minimum copies in different individuals in the same group.

All oral and anal swabs from immunized pigs were collected every other day. In the SY18ΔMGF/CD2v-immunized pigs, both oral and anal swabs showed frequent positive results with qPCR in 1 to 4 pigs at the early stage (i.e., within 5 to 9 days) after inoculation, although no viremia was detected in these pigs, and virus isolation was usually successful in some samples, whereas in the SY18ΔI226R-immunized pigs, only weakly qPCR-positive results were occasionally obtained in a few swabs from 1 or 2 pigs at a later stage (i.e., usually 11 days later) in both the low- and high-dose inoculation groups (Fig. 5A and B), and virus isolation was not successful in all the samples. It is possible that few viruses existed to be isolated or that only degraded viral genome segments were left in these samples. Unusually, only one time of virus shedding was detected in one pig inoculated with a high dose of SY18ΔI226R, and 3 times of virus shedding were detected in one pig inoculated with a low dose of SY18ΔI226R; thus, it seems that virus shedding differs from one individual to another. Nonetheless, shedding is a rational phenomenon of a live virus.

Similarly, after challenge, virus shedding was frequently detected, with high viral DNA copy numbers in almost all SY18ΔMGF/CD2v-immunized pigs at an early stage and in some individuals at a late stage, compared to the challenge control group, whereas shedding of ASFV in the SY18ΔI226R-immunized pigs was detected only occasionally in 1 or 2 pigs with occasional and low viral DNA copy numbers in oral swabs and even less in anal swabs, as shown in Fig. 5C and D. When further checked, the shed virus was found to be virulent.

### Virus and viral DNA were cleared in tissues of SY18ΔI226R-inoculated pigs after the disappearance of viremia.

**FIG 6:**
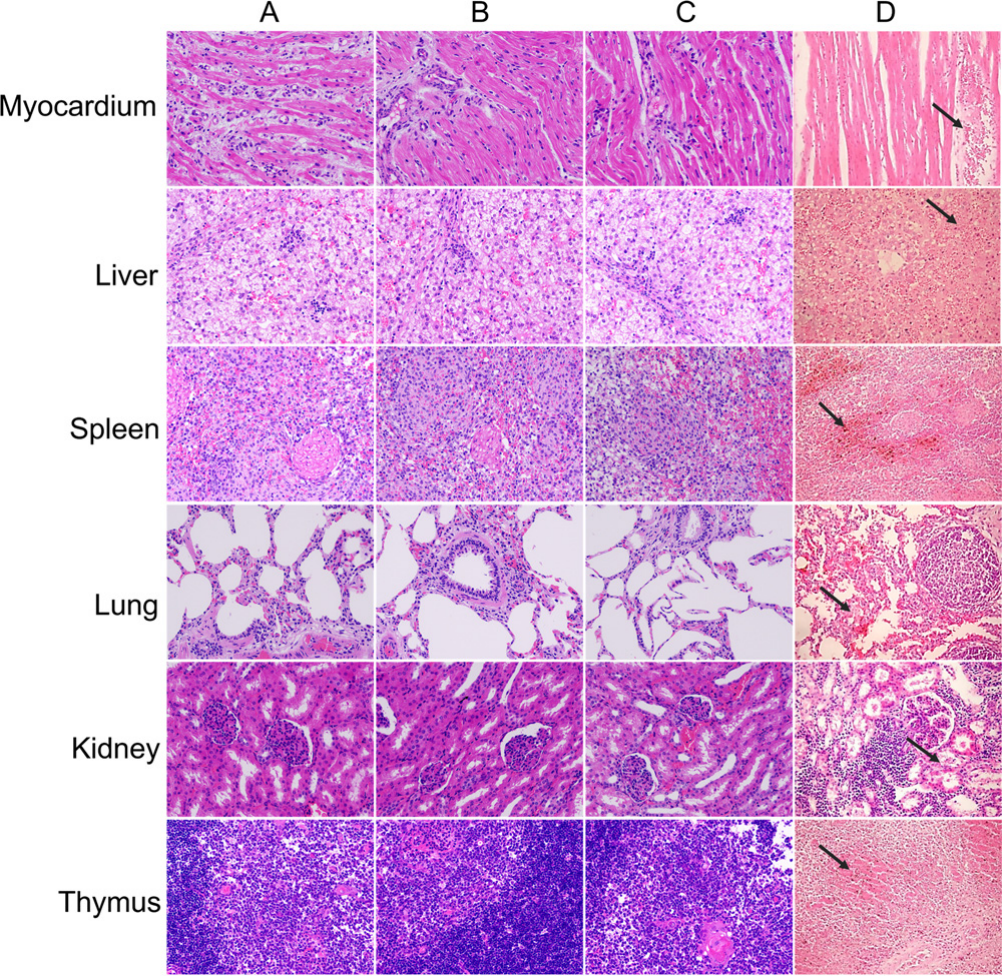
Histopathological sections (hematoxylin and eosin [H&E] staining) of myocardium, liver, spleen, lung, and kidney. In column A, the tissues were collected from pigs inoculated with 10^4.0^ TCID_50_ SY18ΔI226R and challenged with 10^2.5^ TCID_50_ ASFV SY18. In column B, the tissues were collected from the pigs inoculated with 10^7.0^ TCID_50_ SY18ΔI226R and challenged with 10^4.0^ TCID_50_ ASFV SY18. In column C, the tissues were collected from the surviving pigs inoculated with 10^4.0^ TCID_50_ SY18ΔMGF/CD2v and challenged with 10^2.5^ TCID_50_ ASFV SY18. Except for mild vacuolar degeneration in liver samples of all groups (including the challenge control), no other obvious pathological changes are observed in all sections. In column D, the tissues were collected from mock pigs challenged with 10^2.5^ TCID_50_ ASFV SY18. Severe hemorrhage occurred in all sections. Note that the samples from the challenge control (column D) were not stained at the same time as the others, resulting in different color depths between them.

We performed necropsy on all the dead pigs and, when qPCR of the ASFV genome in blood converted to negative, on all the vaccinated and challenge survivors at 28 to 37 dpc in this trial. All organs of the dead pigs in the control group and the SY18ΔMGF/CD2v-immunized group showed typical gross pathogenic changes of African swine fever, such as hemorrhage in the inner organs, while the surviving pigs in both the SY18MGF/CD2v- and SY18ΔI226R-immunized groups showed no pathogenic changes. Histopathological status was observed in major organs or tissues such as the heart, liver, spleen, lung, kidney, and thymus. Except for obvious vacuolar degeneration observed in livers of different vaccinated and surviving pigs, no other histopathological change was found in other organs or tissues, as indicated in Fig. 6.

The qPCR results for ASFV-specific nucleotide in the lung, pulmonary hilar lymph node, bone marrow, and adrenal (gland) and joint fluid in SY18ΔMGF/CD2v-immunized pigs were positive, with high copy numbers (10^3.3^ to 10^8.5^ copies/ml) of viral DNA in the pigs inoculated with SY18ΔMGF/CD2v and even higher copy numbers (10^4.5^ to 10^8.9^ copies/ml) in all the tissues of the pigs in the challenge control group. Differently, the qPCR results were all negative in all the tissues of SY18ΔI226R-immunized pigs in both the low- and high-dose groups, as shown in Fig. 5E, which means that the I226R gene-deleted virus and the challenge virus were all completely cleared from the bodies of the pigs, indicating that transmission-stopping immunity had been produced in the SY18ΔI226R-immunized pigs.

### SY18ΔI226R induced a strong ASFV-specific antibody response.

**FIG 7:**
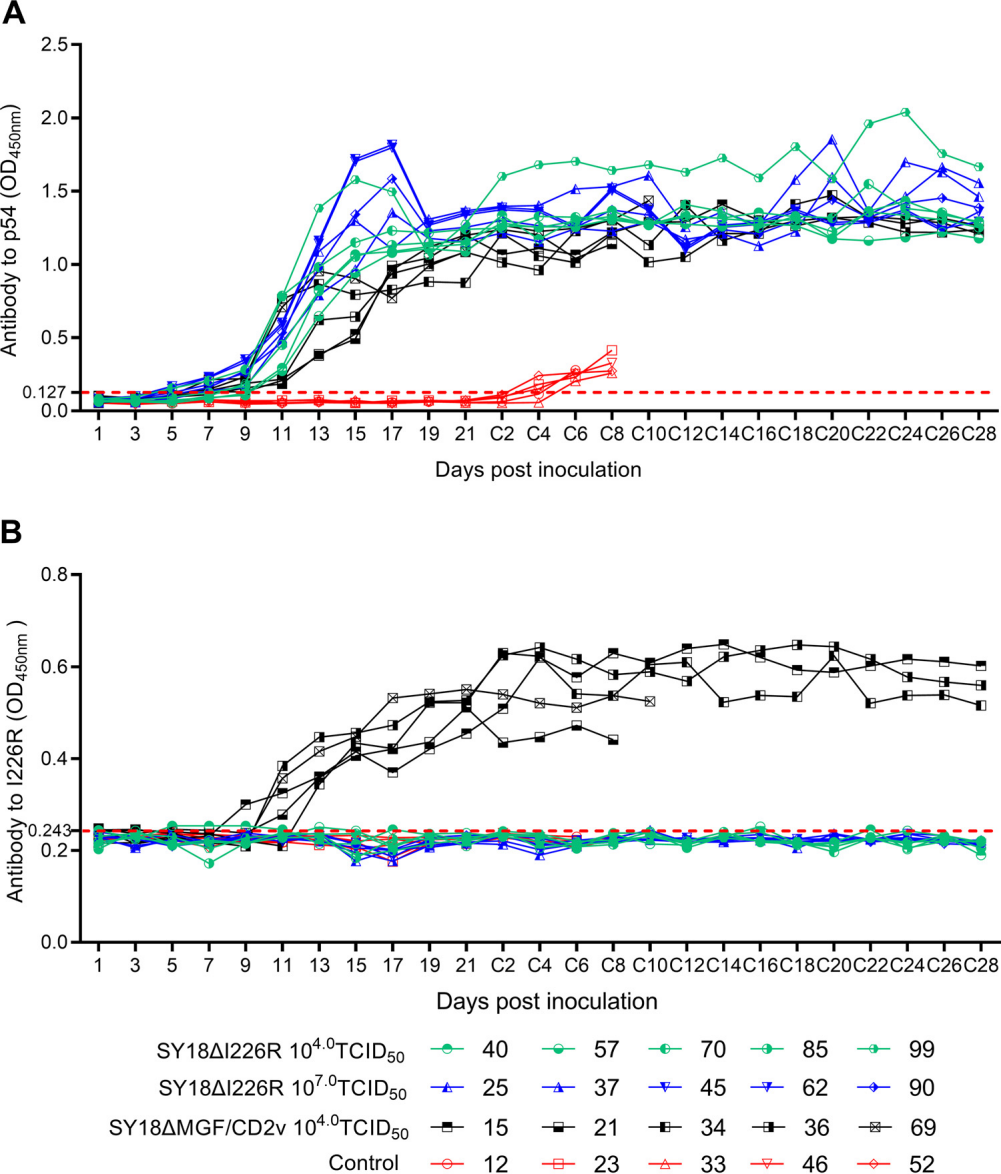
Antibody response curves of different groups of pigs after inoculation and challenge. (A) Antibody to p54. (B) Antibody to I226R.

We tested 3 kinds of antibodies, i.e., p54, pI226R, and the neutralization-like antibody. p54 antibody production conformed to the regular pattern of humoral immune responses of the other attenuated ASFVs (23, 28) and other kinds of viral vaccines (29). The antibody level was detectable at day 9 after inoculation, peaked at around 15 to 17 days postinoculation, and was maintained until the end of the experiment. pI226R antibodies were detected in SY18ΔMGF/CD2v-immunized pigs, and they showed trends similar to that of p54, but the level (optical density at 450 nm [OD_450_] from an indirect enzyme-linked immunosorbent assay [ELISA]) was lower, and the time of antibody production was postponed. After the challenge, the antibodies exhibited a slight increase (Fig. 7A and B).

A neutralization test of sera was performed according to a standard protocol. The results showed that all sera of the 3 different groups did not neutralize ASFV, but a slight inhibitory effect of freshly collected and diluted serum samples on the multiplication of SY18ΔI226R was observed in PAMs when comparing them with repeatedly freeze-thawed serum samples collected from the same individuals. However, the inhibitory effect was transient, and afterwards, virus replication became normal, as usually observed for the fluorescence of the recombinant virus.

## DISCUSSION

The I226R gene with unknown function located at the 3′ end of the ASFV genome encodes a 226-amino-acid protein. There is no significant nucleotide sequence similar to that of I226R and other known genes in the GenBank database; this indicates that there is a typical gene in ASFV. The homology of I226R nucleotide sequences ranges from 94 to 100% among the different ASFV isolates, which indicates that it is a highly conserved, functionally stable, and indispensable gene in ASFV. The I226R gene was expressed in a trend similar to that of the B646L gene during virus proliferation: it showed a very low level of expression at the early stage but increased rapidly to reach the maximum at the middle stage and maintained stability at the late stage. In general, the early-expressed protein of a virus is usually related to virus infection and replication (30, 31), while the late-expressed protein is considered to be related to virus virulence and cellular damage and is closely linked to virus invasion (32). Whether the early expression silencing of the I226R gene plays an important role in ASFV spread at an early stage, to prevent the virus from producing a toxic effect on cells that is conducive to virus replication, diffusion, and proliferation, should be further addressed.

The I226R gene deletion strain SY18ΔI226R infects and replicates efficiently in primary swine macrophage cells *in vitro*. But comparing it with its parental strain SY18, the yield of SY18ΔI226R is observed to be affected, and the level of the final product becomes much lower, indicating that I226R is important for proliferation but not necessarily virus infection and replication. pI226R was observed to be located at the viral factory in the subcellular localization assay; it is possibly a component of the viral particle, so its deletion may harm its growth characteristics *in vitro*. In addition, the CPE on SY18ΔI226R-infected cells appears earlier and is more apparent than with the parental virus SY18, which leads to earlier cell abscission and further affects the multiplication of the virus.

After the inoculation of SY18ΔI226R into pigs, a moderate to high level of viremia was detected, which indicated that the I226R gene had no significant effect on viral replication *in vivo*. However, viremia decreased continuously *in vivo* during 4 weeks of observation, and with the decrease of viremia, a strong specific immune response was elicited, and the virus was gradually cleared from the blood.

Importantly, no abnormal clinical manifestations were observed in all pigs after the inoculation of SY18ΔI226R at either high or low doses. The rectal temperature was maintained in the normal range in all pigs except for an occasional transient rise during the whole observation period. Other pathological changes that were not observed are skin ulcer, hemorrhage, and joint inflammation, etc. All the pigs that survived were healthy; this indicates that the virulence of SY18ΔI226R was completely attenuated. However, all the inoculated pigs showed relatively high levels of viremia that lasted for a longer period than in the SY18ΔMGF/CD2v-immunized pigs (17). As observed in previous studies, the presence of long-term viremia or virus persistence is not a rare event in animals inoculated with other attenuated ASFV strains (17, 33, 34). It was considered to be associated with protection against challenge with virulent ASFV. For I177L gene-deleted ASFV, sentinels with close contact with the inoculated pigs were not infected, no viremia developed, and ASFV antibody was not produced (17). But in our animal experiment, occasional and very low levels of virus shedding were detected in oral and anal swabs by qPCR, but 2 successive positive results were seldom obtained. Whether virus shedding would cause horizontal transmission from inoculated to naive animals needs to be further confirmed in a large-scale animal inoculation trial and with in-contact observation. Nonetheless, virus replication and shedding are the main characteristics of a live vaccine, and if the vaccine is safe, it should favor all sensitive hosts.

Next-generation sequencing showed that three exceptional mutations occurred in the genome of SY18ΔI226R, and two of these mutations did not change the ORF or the peptide length: one is a 7-base insertion in the intergenic region between E423R and E301R, and the other is a single-base mutation in the E199L ORF as a result of an amino acid conversion from Glu to Val. Virulence attenuation associated with these mutations has never been reported. It was noted that in the MGF360-11L ORF, the absence of base C at position 26716 of the SY18 genome led to the truncation of the peptide from 353 to 89 amino acids. Multigene families (MGFs) usually function as interferon inhibitors (34). However, the reported single deletion of an MGF360-1L gene did not reduce the virulence of the strain Georgia2007 (35). The simultaneous deletion of the MGF360-13L and −14L genes in Georgia2007 did not affect its virulence either (36). The single deletion of the MGF110-9L gene in ASFV CN/GS/2018 decreased its replication and reduced its virulence in pigs when given in a low dose (37). For multiple MGF360 genes, natural or artificial deletions have been reported to significantly attenuate the virulence of ASFV isolates, as mentioned above. One typical example is that Reis et al. destroyed the function of nine genes associated with MGF360 and MGF505 of ASFV Benin 97/1, including the MGF360-11L gene, and the resultant BeninΔMGF strain was almost attenuated, with slight residual virulence, thereby causing short transient fever (38). We do not know if the truncation of the MGF360-11L peptide would influence its function, and we also could not predict if the partial deletion of the MGF360-11L protein would affect the virulence of the virus, but the possibility of a cocontribution of MGF360-11L and I226R to the loss of the virulence of SY18ΔI226R could not be excluded.

ASFV-specific antibodies were detected using coated p54 or pI226R antigens; anti-p54 antibody appeared at 9 days postinfection (dpi) and peaked at 15 to 17 dpi. The time of seroconversion of SY18ΔI226R-immune pigs is similar to that for other gene-deleted live ASFV vaccine candidates, although the level of antibody against pI226R was not as high as that of the antibody against p54. The antibody against pI226R was confirmed to be absent in SY18ΔI226R-immunized pigs using an indirect ELISA, which makes the marker to differentiate the SY18ΔI226R vaccine candidate from pigs infected with wild strains and vaccine strains without a deletion of the I226R gene.

Our previous study demonstrated that the intramuscular (i.m.) injection of 10^2.5^ TCID_50_ of virulent SY18 could cause death of some of the pigs inoculated with two shots of 10^4.0^ TCID_50_ of SY18ΔMGF/CD2v, and intramuscular injection of 10^4.0^ TCID_50_ of SY18 could cause all pigs inoculated with 10^4.0^ TCID_50_ of SY18ΔMGF/CD2v to succumb to death, but the oral administration of SY18 could not cause any death in the vaccinated pigs (18); this reflects the importance of mucosal immunity to ASFV. However, all the SY18ΔI226R-inoculated pigs survived a high-dose intramuscular injection challenge with SY18, and all clinical presentations remained normal except for a transient and mild temperature rise for 1 or 2 days. All the results demonstrated that SY18ΔI226R is a highly effective candidate ASF vaccine that can protect inoculated pigs against virulent virus challenge through injection.

Virulence determinants of ASFV include several aspects due to the intricacy of virus particle composition. For example, ASFV can inhibit host interferon production using products of MGFs (34, 38, 39), DP96R (40) and I329L (41), and it can also inhibit stress and cell apoptosis using DP71L (27, 42). The virus may have other functions that directly or indirectly influence its virulence in pigs. Recently, the I177L gene was identified to be a key element in determining the virulence of ASFV. After its deletion, ASFV-G-ΔI177L did not cause any clinical abnormality and provided pigs full protection against challenge with its parental strain, although the functional mechanism of the I177L gene of ASFV has not yet been recognized (17). Similarly, we reported the discovery of a newly recognized gene, I226R, that determines the virulence of ASFV. Its deletant variant, denoted SY18ΔI226R, exhibited very exciting characteristics, including having no side effects *in vivo*, producing acceptable viremia, and eliciting robust immune responses. The mechanisms of its virulence attenuation and immune protection remain to be clarified. We speculated that the genes I226R and I177L or their products may be an important switch or a key link in a cascade reaction to maintain the pathogenicity of ASFV in pigs, as when I226R was deleted, I177L was present in the genome and vice versa. However, the detailed mechanism needs further study.

Viremia was apparent after inoculation with SY18ΔI226R and challenge with SY18. However, with the antibody produced, viremia was gradually reduced, and when viremia disappeared, the necropsied tissues were negative for ASFV by qPCR; this indicated that the attenuated virus or virulent SY18 was completely cleared from the body, and it seemed that transmission-stopping immunity was produced in the inoculated pigs. This phenomenon was similar to that found with ASFV-G-ΔI177L (17). We assayed only the level of antibody but did not detect changes of cytokines in immune pigs. Regardless of whether immune pig serum plays a role in viral inhibition, clearance is still worth further investigation.

In summary, our study suggests that the I226R gene of ASFV is a newly identified key gene in determining virulence, and the deletion of this single gene completely attenuates the virulence of SY18 in swine. Furthermore, inoculation with the deletant strain SY18ΔI226R provides robust immunity and protects vaccinated pigs from intramuscular challenge with the virulent parental strain SY18, even at a high dose. All our results demonstrate that SY18ΔI226R is a promising attenuated vaccine candidate for ASF prevention.

## MATERIALS AND METHODS

### Cells and viruses.

The pulmonary alveolar macrophages (PAMs) used in this study were prepared from 2- to 3-month-old piglets. Briefly, the intact lung was filled with sterilized phosphate-buffered saline (PBS), and we then collected the bronchoalveolar lavage fluid. Cells were collected via centrifugation at 560 × *g* for 10 min and resuspended using PBS. After removing the erythrocytes and rinsing with PBS, the cell pellets were resuspended, and the cells were grown in RPMI 1640 (Gibco) supplemented with 10% fetal bovine serum (Gibco). Cells were cultured in an incubator at 37°C under 5% CO_2_. We performed nucleic acid testing according to China national standards to ensure that there was no contamination with viruses infecting swine, which include ASFV, classical swine fever virus (CSFV), porcine reproductive and respiratory syndrome virus (PRRSV), pseudorabies virus (PRV), porcine parvovirus (PPV), and porcine circovirus 1/2 (PCV1/2).

The ASFV SY18 strain (GenBank accession no. MH766894), a field ASFV isolate, was isolated from pig specimens after the first outbreak of ASF in China in August 2018 by our laboratory, which caused acute infection in pigs and resulted in up to 100% fatality in the farm. The virus was passaged in primary PAMs and stored at −80°C. The SY18ΔMGF/CD2v strain, an attenuated strain of ASFV bearing deletions of the MGF505-1R, MGF505-2R, MGF505-3R, MGF360-12L, MGF505-13L, MGF505-14L, and EP402R ORFs through homologous recombination, was also used in this study for comparison in vaccination and challenge trials. It has been proven to protect vaccinated pigs from virulent isolate SY18 infection when challenged by the oral administration of 10^3.0^ TCID_50_ (18).

ASFV strain SY18 was titrated using 96-well plates and observed by an immunofluorescence assay, with staining with a fluorescein isothiocyanate (FITC)-labeled monoclonal antibody against the ASFV protein p30. Briefly, virus suspensions were 10-fold diluted and inoculated onto the cell monolayer. After incubation for 5 days at 37°C, the cultures were fixed using 80% cold acetone and incubated with the FITC-labeled p30 monoclonal antibody (prepared in our laboratory) for 1 h at 37°C, followed by observation under a fluorescence microscope. For the gene-deleted strains, the fluorescence emitted directly from eGFP was observed. The Reed-Muench method was used to calculate the virus titer (43).

### Sequence analysis.

Both the nucleotide and amino acid sequences of the SY18 I226R gene were used for analysis using the PubMed Nucleotide BLAST website (https://blast.ncbi.nlm.nih.gov/Blast.cgi) to compare homology with those of the other ASFV strains and with possible preexisting heterologous genes or proteins. In addition, a multiple amino acid sequence alignment was performed using MAFFT and Jalview software.

### SYBR green I-based quantitative PCR assay.

PAMs in 12-well plates were infected with ASFV SY18 at a multiplicity of infection (MOI) of 3. Uninfected cells were used as controls. Cell samples were collected at 2, 4, 6, 8, 11, 14, 17, and 20 h postinoculation (hpi). An RNA simple total RNA kit (Qiagen) was used to extract the total cellular RNA. The elimination of ASFV genomic DNA and reverse transcription of the total RNA *in vitro* were performed according to the instructions of the PrimeScript RT reagent kit with gDNA Eraser (TaKaRa). The cDNA was detected using primers for SY18 B646L (encoding the p72 protein), CP204L (encoding the p30 protein), I226R, and GAPDH (housekeeping gene) (GenBank accession no. KJ786424.1), based on SYBR green I-based real-time quantitative PCR (qPCR). The primers used in this assay were designed by using Primer Premier 6 software, and the sequences are presented in Table 3. The results were analyzed using GraphPad Prism 8 software.

**TABLE 3:** Primers used for determination of gene expression by qPCR

Gene	Forward primer (5′–3′)	Reverse primer (5′–3′)
B646L	CGAACTTGTGCCAATCTC	ACAATAACCACCACGATGA
CP204L	TTCTTCTTGAGCCTGATGTT	TAGCGGTAGAATTGTTACGA
I226R	CAGGAACTATCACGGCTTAT	CGTAATGTAGAGTCTGCTTATC
GAPDH	CCTTCATTGACCTCCACTACA	GATGGCCTTTCCATTGATGAC

### Indirect immunofluorescence assay.

We performed an indirect immunofluorescence assay according to a method described previously, with minor modifications (44). Briefly, the PAMs were seeded into a 12-well plate at 80% confluence and infected with ASFV SY18 (MOI = 3) 6 h after seeding. After 72 h, the PAMs were washed once and fixed in 80% cold acetone for 20 min at 4°C. Next, the wells were blow-dried, and rabbit anti-pI226R serum and mouse anti-p54 serum antibodies (prepared in our laboratory) were diluted 1:50 and 1:200, respectively, in PBS and incubated together for 1 h at 37°C. The goat polyclonal secondary antibody to rabbit IgG-conjugated TRITC (Beyotime) and the goat polyclonal secondary antibody to mouse IgG-conjugated FITC (Beyotime) were diluted 1:500 in PBS and incubated for 1 h together at 37°C in the dark. Each incubation step described above was interspersed by washes three times. We added antifade mounting medium with DAPI (Beyotime) to the wells. The results were observed under an inverted fluorescence microscope (IX73; Olympus).

### Construction of gene-deleted SY18ΔI226R.

The I226R gene was deleted from the ASFV SY18 genome and replaced with an eGFP gene by homologous recombination between the SY18 virus and a recombinant plasmid comprising the eGFP expression cassette under the control of the p72 promoter and two sequences flanking the I226R gene of about 1,200 bp. The recombination plasmid of about 2 μg was transfected into PAMs in a 12-well plate using jetPEI-Macrophage DNA transfection reagent (Polyplus). After 4 h, the transfected cells were infected with ASFV SY18 at an MOI of 3 and continued to be cultured at 37°C under 5% CO_2_. The cells exhibiting fluorescence were picked out under a fluorescence microscope, further screened, and purified by limiting dilution. The final recombinant virus was amplified in PAMs. The purity of SY18ΔI226R was determined by PCR. The forward and reverse primers 5′-CTACGCCGAACTATTCAGA-3′ and 5′-GTCACCAACAACAGGATAAC-3′ were designed based on the sequences in the middle of the I226R coding region and the right flanking region, respectively, using Primer Premier 6 software. A 492-bp fragment could be amplified if parental SY18 exists.

### Characterization of SY18ΔI226R *in vitro*.

To evaluate the effect of the I226R gene deletion on viral replication *in vitro*, PAMs were infected with purified SY18ΔI226R and parental SY18 at an MOI of 0.01. Next, the culture was collected at 2, 12, 24, 36, 48, 72, 96, and 120 hpi, and freeze-thawing was repeated 3 times. All time points were repeated three times. The TCID_50_s of the recombinant virus and SY18 at different time points were assayed by lysis of the liquid-nitrogen-freeze-thawed virus cell culture.

### Next-generation sequencing.

Both the SY18ΔI226R and SY18 viral genomic DNAs were extracted from the infected PAMs using a body fluid viral DNA/RNA miniprep kit (Axygen, USA). The full-length sequence of the modified mutant ASFV genome was determined by next-generation sequencing (NGS) using the Illumina novaseq6000 PE150 platform (Novogene, Tianjin, China).

### Animal experiments.

All animal experiments were approved by the Animal Welfare and Ethics Committee of Changchun Veterinary Research Institute under IACUC approval number AMMS-11-2019-001 (approved 30 August 2019), and the ASFV cultivation and animal experiments were conducted in an animal biosafety level 3 (ABSL-3) laboratory.

To evaluate the effect of the I226R gene deletion on the virulence of ASFV SY18 *in vivo*, a total of 20 Landrace pigs free of both ASFV antigen and antibody were clustered randomly into 4 groups with 5 pigs in each group. The first two groups were inoculated intramuscularly (i.m.) with 10^4.0^ TCID_50_ and 10^7.0^ TCID_50_ of SY18ΔI226R, respectively. The third group was inoculated with 10^4.0^ TCID_50_ of SY18ΔMGF/CD2v. The last group was set as the control for challenge. The rectal temperature was measured every day. Oral and anal swabs and blood samples were collected every other day for viral nucleic acid detection via probe-based qPCR or antibody assays by an ELISA. Clinical symptoms such as high fever, inappetence, depression, diarrhea, waddling, reluctance to stand, skin cyanosis, and arthrocele were observed and recorded daily throughout the experiment. The pigs showing severe clinical signs were euthanized *in extremis* using pentobarbital, and the tissues, including submaxillary lymph nodes, tonsil, heart, lung, thymus, marrow, liver, spleen, kidney, stomach, colon, jejunum, bladder, inguinal lymph nodes, joint fluid, and muscle, were collected for viral load assessment via qPCR.

On day 21 postinoculation, pigs inoculated with 10^7.0^ TCID_50_ of SY18ΔI226R were challenged by intramuscular injection with 10^4.0^ TCID_50_ of SY18, while the other pigs were challenged by intramuscular injection with 10^2.5^ TCID_50_ of SY18. Clinical observation, sampling, and detection were performed as mentioned above.

When viremia disappeared, the pigs were necropsied to check for gross tissue lesions, including hemorrhage, lymphadenopathy, necrosis, effusion, and fibrosis. The tissues and organs were collected in order to detect ASFV nucleic acid via qPCR and fixed in paraformaldehyde for histopathological analysis.

### Quantification of the ASFV load.

Probe-based qPCR targeting the ASFV B646L gene (encoding the p72 protein) was performed to determine the ASFV load in the blood and tissues. Primer synthesis and reaction conditions were the same as those recommended by the World Organization for Animal Health (OIE) (22). qPCR was performed using Premix Ex *Taq* (probe qPCR) reagent (TaKaRa) and Eco (Illumina). Copy numbers of the ASFV genome samples were calculated according to the standard curve established by the detection of the standard plasmid containing the ASFV SY18 B646L gene (p72).

### Indirect ELISA.

The ASFV-specific antibodies targeting p54 and pI226R were assayed using the indirect ELISA method. Serum samples from a naturally ASF-recovered pig and a negative pig were used as positive and negative controls. Taking the assay of p54 antibodies as a case study, the detailed process was described in a previous study (26). Briefly, ELISA plates (Corning) were coated with purified p54 protein at 4°C overnight and blocked with 5% skimmed milk at 37°C for 2 h. The serum sample, positive control, and negative control were added to the ELISA plates and incubated for 1 h at room temperature (RT). Horseradish peroxidase (HRP)-labeled sheep anti-pig IgG, used as the secondary antibody, was incubated for 1 h at RT. The chromogenic reaction began with the addition of 3,3',5,5'-tetramethylbenzidine (TMB) substrate and ended with 2 M sulfuric acid. The optical density at 450 nm (OD_450_) values were read using an iMark microplate reader (Bio-Rad, USA). An OD_450_ value above 0.243 (the average OD_450_ plus 3 standard deviations [SD] from 100 negative serum samples) is considered positive. In addition, the antibody targeting pI226R was detected using the same method. The antigens of p54 and pI226R were prepared using a prokaryotic expression system in our laboratory. The OD_450_ values were analyzed using GraphPad Prism 8.0.

### Neutralization test.

Testing of serum samples, including those from SY18ΔI226R-immuned pigs, an ASF-recovered pig, and a negative pig, was performed using a 2-fold gradient dilution with RPMI 1640 medium. The primary serum and dilutions were mixed with the same volume of SY18ΔMGF/CD2v (100 TCID_50_). Simultaneously, SY18ΔMGF/CD2v plus the negative serum sample were set as positive controls, and RPMI 1640 medium plus the negative serum sample were set as negative controls. All samples were tested in duplicate and incubated at 37°C for 1 h. Next, PAMs were added to a 96-well plate (2 × 10^5^ cells/well) and cultured at 37°C under 5% CO_2_. The change in fluorescence was observed under a fluorescence microscope from 2 dpi to 5 dpi.

### Statistical analysis.

Statistical significance was determined using the Holm-Sidak test, and a *P* value of <0.05 was considered statistically significant. Similar results were obtained from three independent experiments. Statistically significant differences between groups were analyzed using GraphPad Prism 8.0 software (GraphPad, La Jolla, CA).
